# Bacteraemia variation during the COVID-19 pandemic; a multi-centre UK secondary care ecological analysis

**DOI:** 10.1186/s12879-021-06159-8

**Published:** 2021-06-11

**Authors:** Sarah Denny, Timothy M. Rawson, Peter Hart, Giovanni Satta, Ahmed Abdulaal, Stephen Hughes, Mark Gilchrist, Nabeela Mughal, Luke S. P. Moore

**Affiliations:** 1 Chelsea and Westminster NHS Foundation Trust, 369 Fulham Road, London, SW10 9NH UK; 2 Imperial College Healthcare NHS Trust, Praed Street, London, W2 1NY UK; 3 Department of Infectious Diseases, Imperial College London, Hammersmith Campus, Du Cane Road, London, W12 0NN UK; 4 Independent Researcher, London, UK; 5 North West London Pathology, Fulham Palace Road, London, W6 8RF UK

**Keywords:** Microbiology, Blood culture, Coronavirus, SARS-CoV-2

## Abstract

**Background:**

We investigated for change in blood stream infections (BSI) with Enterobacterales, coagulase negative staphylococci (CoNS), *Streptococcus pneumoniae,* and *Staphylococcus aureus* during the first UK wave of SARS-CoV-2 across five London hospitals.

**Methods:**

A retrospective multicentre ecological analysis was undertaken evaluating all blood cultures taken from adults from 01 April 2017 to 30 April 2020 across five acute hospitals in London. Linear trend analysis and ARIMA models allowing for seasonality were used to look for significant variation.

**Results:**

One hundred nineteen thousand five hundred eighty-four blood cultures were included. At the height of the UK SARS-CoV-2 first wave in April 2020, Enterobacterales bacteraemias were at an historic low across two London trusts (63/3814, 1.65%), whilst all CoNS BSI were at an historic high (173/3814, 4.25%). This differed significantly for both Enterobacterales (*p* = 0.013), CoNS central line associated BSIs (CLABSI) (*p* < 0.01) and CoNS non-CLABSI (*p* < 0.01), when compared with prior periods, even allowing for seasonal variation. *S. pneumoniae* (*p* = 0.631) and *S. aureus* (*p* = 0.617) BSI did not vary significant throughout the study period.

**Conclusions:**

Significantly fewer than expected Enterobacterales BSI occurred during the UK peak of the COVID-19 pandemic; identifying potential causes, including potential unintended consequences of national self-isolation public health messaging, is essential. High rates of CoNS BSI, with evidence of increased CLABSI, but also likely contamination associated with increased use of personal protective equipment, may result in inappropriate antimicrobial use and indicates a clear area for intervention during further waves.

**Supplementary Information:**

The online version contains supplementary material available at 10.1186/s12879-021-06159-8.

## Background

Severe Acute Respiratory Syndrome coronavirus 2 (SARS-CoV-2) began in December 2019 in China. Transmission within Europe, including the United Kingdom (UK), was confirmed by the end of February 2020. In the UK, social distancing and self-quarantine measures were subsequently implemented, aimed at slowing transmission. Furthermore, in an attempt to prevent overwhelming hospital capacity, those with high temperature and/or new continuous cough (i.e. symptoms consistent with COVID-19, but also other infections) were advised to stay home and seek advice online or via a national telephone service [1]. Elective clinical services were also reduced, with cancellation of non-urgent clinics and surgery. Extensive self-isolation practices and decreased utilisation of healthcare services has potentially impacted on direct care for both COVID-19 and non-COVID-19 clinical presentations.

Altered presentation of bloodstream infections (BSI) to healthcare is one potential indirect consequence of national public health measures. Whilst some organisms associated with BSIs have a clear communicable component (for example *Streptococcus pneumoniae*, *Neisseria meningitidis* and *Staphylococcus aureus*) [2–4], others are likely to arise endogenously [5]. Therefore, the incidence of communicable pathogens may decrease secondary to social distancing, or increase due to increased time spent with household contacts, while incidence of likely endogenous pathogens, such as *Escherichia coli* and other Enterobacterales, should be less affected. Potential changes must be considered however in the context of the seasonality for Enterobacterales (peaks seen in summer) [6, 7] and *S. pneumoniae* (peaks in winter). Finally, coagulase negative staphylococci (CoNS) are skin commensals and frequent contaminants of bloods cultures, although they can be implicated in BSIs for example, in the presence of intravascular catheters. Lower rates are seen with increased phlebotomy expertise/appropriate skin decontamination. International targets for contamination are less than 3% [8]. It may be hypothesised that incidence would increase with changes to working conditions, including implementation of personal protective equipment (PPE) for staff.

With NHS England data suggesting that emergency presentations to secondary care were down 29.4% in March 2020 compared to March 2019 [9], there are concerns over delayed or missed presentations with non-COVID-19 infections. To explore this, we investigated the incidence of BSIs during the initial months of the COVID-19 pandemic, focusing on Enterobacterales and CoNS, against variations in rates across the preceding 3 years. We used Enterobacterales, *S. pneumoniae*, and *S. aureus* BSI as indicator organisms for missed bacteraemia presentations and CoNS BSIs not related to a central line associated BSI (non-CLABSI) as a surrogate for contamination.

## Methods

### Study setting and design

A retrospective multicentre ecological analysis was undertaken evaluating all blood culture (BC) samples from adults (aged 17 years and above) over a three-year period from 01 April 2017 to 30 April 2020 across five acute hospitals in London, serving a population approximating 3 million.

A hub-and-spoke laboratory network with a centralized microbiology laboratory processes samples from multiple hospitals in accordance with UK laboratory standard operating procedures [10] with minor local variation. BCs were collected at each hospital, without pre-incubation, and transported to the centralized laboratory. They were subsequently incubated using a BACTEC system (Becton Dickinson, Franklin Lakes, NJ, USA). Organisms were identified by matrix assisted laser desorption/ionisation-time-of-flight (MALDI-TOF) mass spectroscopy (Bruker Daltonik GmbH, Bremen, Germany). Susceptibility testing was undertaken using disk diffusion using European Committee on Antimicrobial Susceptibility testing methods and interpretative criteria [11].

### Data collection

Microbiological data was extracted from Sunquest Laboratory V8.3 (Tucson, AZ, USA). For the purposes of this study, Enterobacterales included *Escherichia coli*, *Klebsiella* spp., *Serratia* spp., *Enterobacter* spp., *Proteus* spp., *Citrobacter* spp., *Hafnia* spp., *Morganella* spp., and *Pantoea* spp. All CoNS, *S.pneumoniae* and *S.aureus* isolated from BCs were included; the seasonality of the latter two has been extensively described [12–15]. If more than one pathogen was isolated from a BC, each was recorded individually at either genus or species level. Samples with multiple Enterobacterales or CoNS were recorded as a single positive BC. Repeated positives within a 14-day period were de-duplicated except4 for CoNS related to CLABSIs. In order to determine this, blood patient notes were accessed and those with repeated BSIs with the same CoNS within a 14 day period in the presence of a central venous catheter, were classified as CLABSIs. Those where this could not be determined were not included in the statistical analysis.

### Data analysis

To explore seasonal variations in BSI rates, results were classified as spring (March–May), summer (June–August), autumn (September–November) and winter (December–February). For spring 2017, only April and May data were available. March and April 2020 were considered as the period when social distancing measures were in place, encompassing the UK COVID-19 peak [16]. The same period in 2019 was analysed for comparison.

To explore changing trends in BCs results during COVID-19, two different statistical models were used; both (first) allowing for seasonality, and (second) then separately obviating any potential seasonality. The first method was a time-series analysis performed in R (R Core Team). After testing for the absence of first-order autocorrelations with the Durbin-Watson statistic, univariable autoregressive integrated moving average (ARIMA) models were fitted to data from CoNS (non-CLABSI and CLABSI), Enterobacterales, *S.pneumoniae*, and *S.aureus* from April 2017 to December 2019. These models were used to forecast estimated BSI rates for January to June 2020. To explore differences between observed and expected BSI rates during the COVID-19 peak, they were graphically compared.

The second method was a linear trend analysis of variations in observed rates of BSIs. Z-scores were calculated for Enterobacterales, CoNS (non-CLABSI and CLABSI), *S.pneumoniae* and *S.aureus* using SPSS (IBM Corp, Armonk, NY, USA). Data were checked for normality using the Shapiro-Wilk test. Where non-normal distribution was identified, data were transformed prior to analysis. Z-scores were then calculated each month by applying the standard formula z = (x-μ)/σ (x = raw score, μ = mean, σ = standard deviation). Statistical significance for z-scores were determined using R (R Core Team).

### Study approval

This analysis was registered with North West London Pathology hosted by Imperial College Healthcare NHS Trust as a service evaluation (reference PAT_012) to investigate BC contamination rates. Individual consent was not indicated for this ecological level analysis reporting only aggregated data.

## Results

During April 2017 to April 2020, 133,856 BCs were identified. Of those, 12,625 were from an external source (non-networked hospital), 2002 unclassifiable, and 729 not processed, leaving 119,584 for analysis. The overall number of BC requests remained stable throughout the entire period in terms of absolute numbers. From the 119,584 BCs, no pathogen/microorganism was detected in 109,144 (91.27%). Growth was demonstrated in 10,440 cultures (8.73%). Enterobacterales were the most commonly isolated pathogen (3508 cultures, 2.93% of total), followed by CoNS (2855 cultures, 2.39%). Full details of cultured microorganisms and number of tests are provided (Table 1).

**Table 1:** Summary of positive and negative blood cultures collected during the period April 2017 to April 2020 across a six London hospital network

	Group	Number of blood cultures	% of total blood cultures
Blood cultures with no growth (N; % of all blood cultures)		109,144	91.27%
Blood cultures with isolate identified (N; % of all blood cultures)	CoNS all	2855	2.39%
	- CLABSI	2466	2.06%
	- Non-CLABSI	341	0.29%
	- Unknown	48	0.04%
	S.pneumoniae	78	0.07%
	GNR-E	3508	2.93%
	GNR-NE	790	0.66%
	Other	1975	1.65%
	S.aureus	988	0.83%
	Yeasts	246	0.21%

*Abbreviations: CoNS* coagulase negative staphylococci, *CLABSI* Central line associated blood stream infection, *Non-CLABSI* Non-central line associated blood stream infection, *Unknown* unable to classify if CLABSI or Non-CLABSI, *S.pneumoniae Streptococcus pneumoniae, GNR-E* Gram Negative Rods – Enterobacterales, *GNR-NE* Gram Negative Rods- Non Enterobacterales, *Other* including Group A/B/C/G Streptococci, Gram positive rods, *Actinobacillus spp*., *Aerococcus spp*., *Microbacterium spp*., *Micrococcus spp, S.aureus Staphylococcus aureus* (both meticillin susceptible and meticillin resistant), *Yeasts predominantly Candida spp.*

### Seasonal trends

**Fig. 1:**
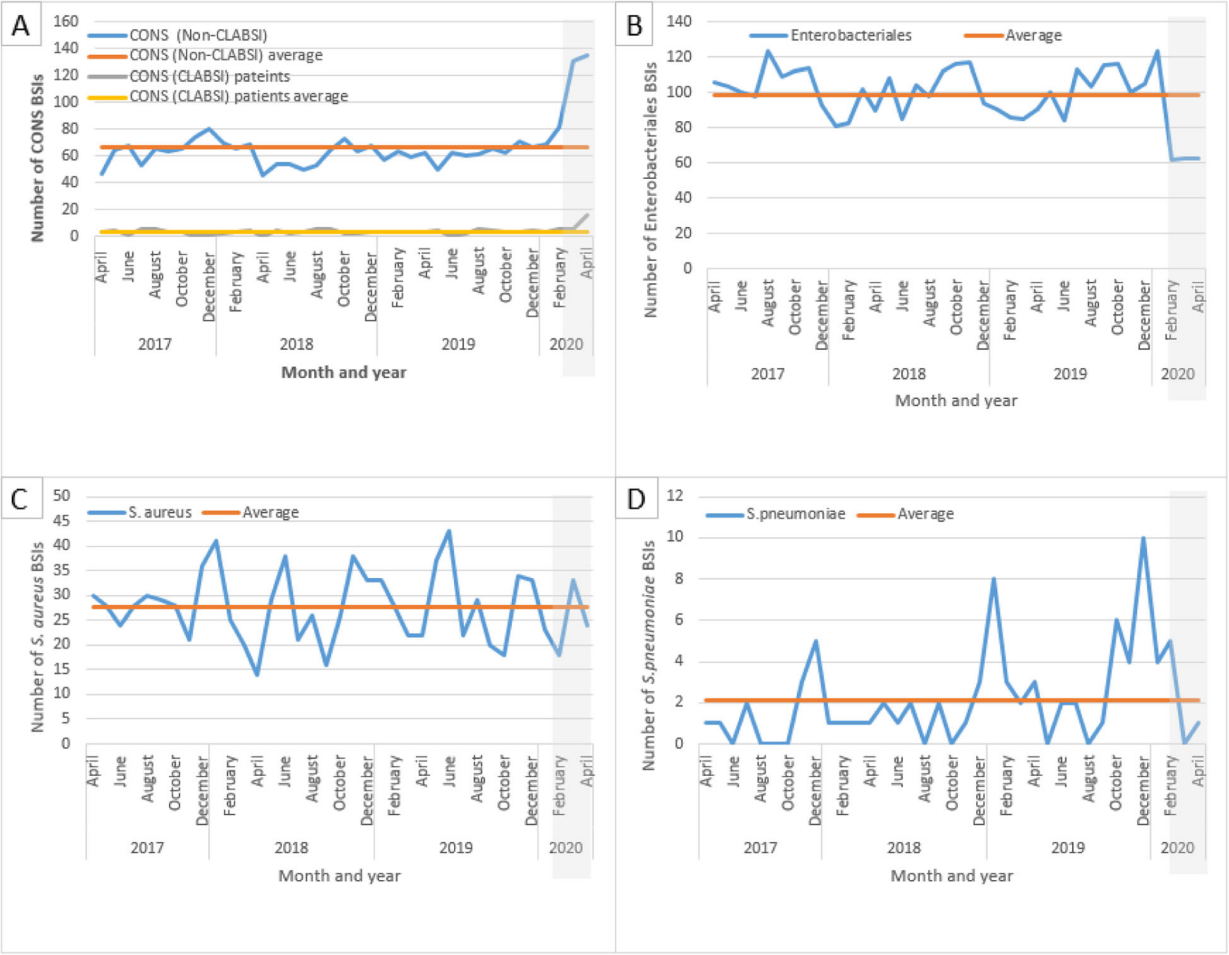
Comparison of observed blood stream infection with linear trend analysis during the period April 2017 to April 2020 across a six London hospital network for **a** coagulase negative staphylococci, **b** Enterobacterales, **c** *S.aureus*, and **d** *S.pneumoniae*

**Fig. 2:**
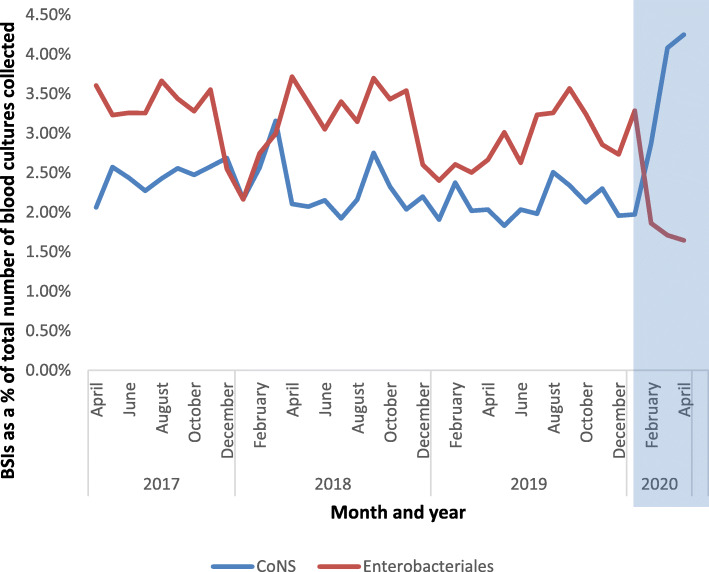
Change in blood culture isolates across six London hospitals during the COVID-19 social isolation measures in March and April 2020. Enterobacterales and CoNS (total) are shown and expressed as a percentage of the total number of blood cultures collected. For breakdown of CONS (non-CLABSI and CLABSI), please see Fig. 1

Seasonal trends were observed for *S.pneumoniae* and Enterobacterales. The seasonality of *S.pneumoniae* (preponderance towards winter months) is shown in Fig. 1d, whilst the seasonality of Enterobacterales (peaks in summer months) is shown in Fig. 1b. Across the cohort, Enterobacterales as a percentage of all BCs taken was lowest in the winter (2.49% 2017, 2.54% 2018 and 2.63% 2019), with a general trend towards sequentially rising throughout spring and into summer before peaking in autumn (3.42% 2017, 3.56% 2018 and 3.22% 2019) (Fig. 2). For all CoNS the mean percentage over the study period is 2.39%. There was little seasonal variation prior to COVID-19 (Fig. 2). For *S. aureus* the linear mean over the study period was 27.57, with significant variation over time, but little evidence of recursive seasonality (Fig. 1c).

### Bacteraemia variation during the UK COVID-19 peak March and April 2020

In April 2020, Enterobacterales as a percentage of all BCs taken were at their lowest during the study period at 1.65% (second lowest March 2020 at 1.71%; Fig. 2). In contrast, all CoNS, as a percentage of all BCs taken, were at their highest during the study period at 4.25% (second highest in March 2020 at 4.08%) (Fig. 2).

The linear trend analysis (Table 2**)** for Enterobacterales, CoNS, *S.aureus* and *S.pneumoniae* from April 2017 to April 2020 suggests significant differences in BSI rates, particularly during the COVID-19 period. Enterobacterales BSIs were significantly lower than the mean in February, March, and April 2020 only (Z-scores; − 2.57, − 2.48, and 2.48, respectively; *p* < 0.05). In contrast, CoNS (non-CLABSI) BSIs were significantly above the mean for March and April 2020 (Z-scores; 3.56, 3.78 respectively; *p* < 0.01). Similarly, CoNS (CLABSI patients) were significantly above the mean in April 2020 (Z-score 4.71, *p* < 0.01). Numbers detected did not deviate significantly from the mean from April 2017 until February and March 2020, respectively for these organisms. *S.aureus* BSIs did not vary significantly from mean throughout the period analysed. *S.pneumoniae* BSIs did not vary significantly from the mean throughout the period analysed, except for in December 2019. Before Spring 2020, the highest number of patients with a CLABSI was six per month (range 0–6), however, in April 2020 we identified 16 patients with a CLABSI. Whilst the numbers were too small to undertake statistical analysis, there was a trend towards an increased number of patients with CLABSIs in April 2020.

**Table 2:**
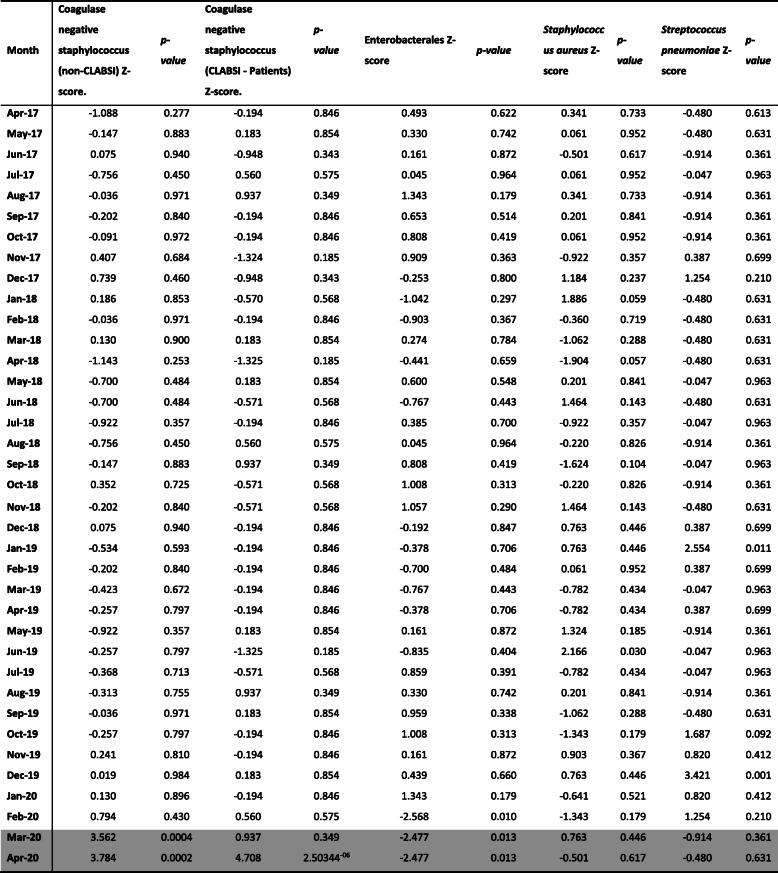
Variation in blood stream infection during the period April 2017 to April 2020 across a six London hospital network for Enterobaterales, CoNS, S*.aureus* and *S.pneumoniae*. Z-score calculated as variation from linear mean. The month of March and April 2020 during the COVID-19 social isolation measures are highlighted in grey

**Fig. 3:**
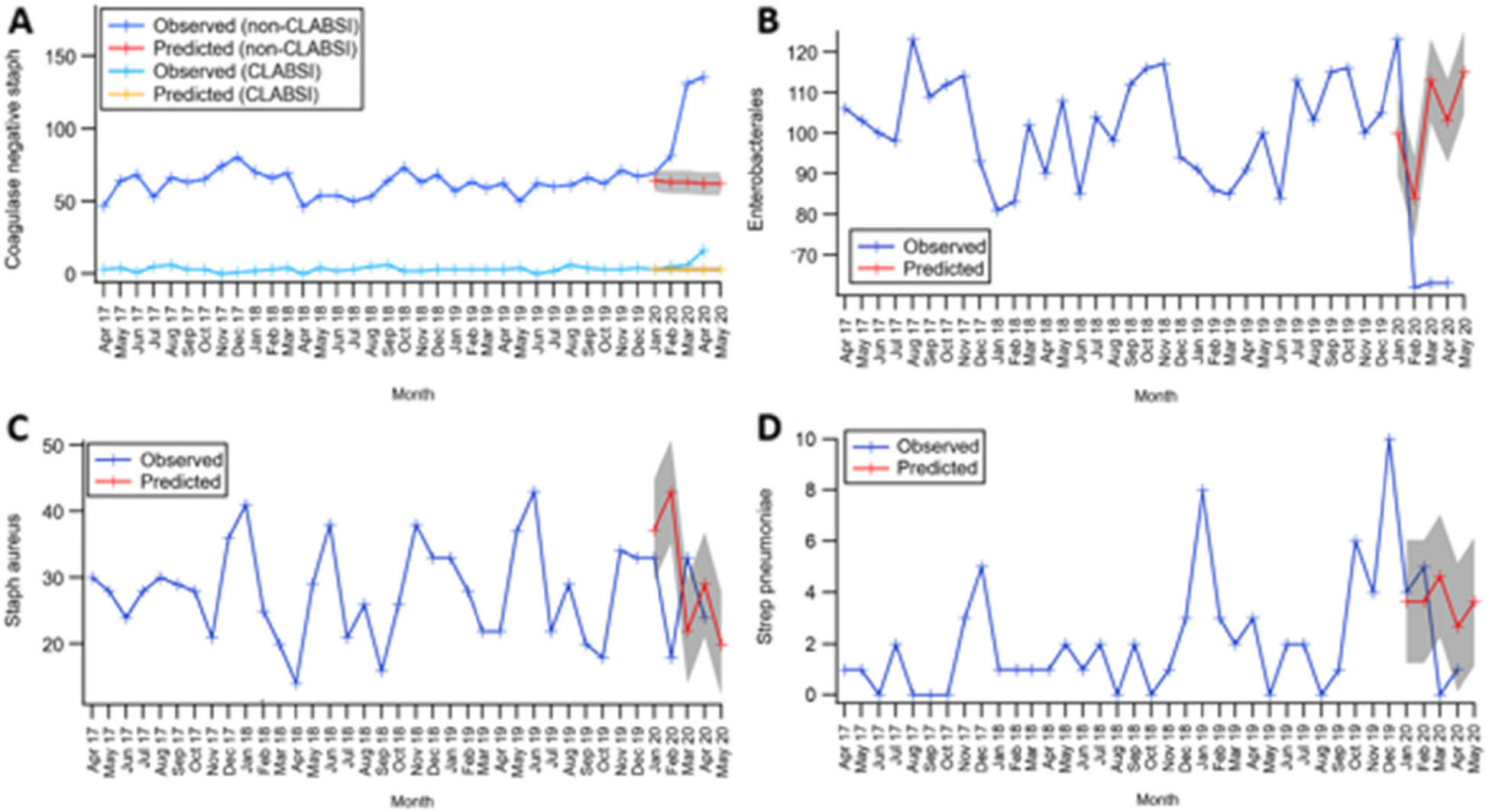
Comparison of observed versus predicted (ARIMA model Jan-June 2020) trends in blood stream infection during the period April 2017 to April 2020 across a six London hospital network for **a** Coagulase negative Staphylococci, **b** Enterobacterales, **c** *S.aureus*, and **d** *S.pneumoniae*

The ARIMA model, constructed to allow for any seasonality, is represented in Fig. 3a-d and demonstrates historic observed BSI (April 2017–December 2019) and then predicted versus observed BSI from January 2020 to June 2020. Once seasonality is allowed for, this also showed a significant divergence in observed versus predicted Enterobacterales and CoNS from February to April 2020. Fewer numbers of Enterobacterales BSIs and higher numbers of CoNS BSIs (non-CLABSI and patients with CLABSIs) were seen. Numbers of *S.aureus* and *S.pneumoniae* BSI remained within the limits of confidence of predicted based on historical trends.

## Discussion

In our multi-centre BSI ecological analysis we find during the period of COVID-19 social distancing and self-isolation a significant reduction in Enterobacterales BSIs verified across two different statistical models. Similarly, there was a significant increase in non-CLABSI CoNS (presumptively contamination) BSI, whilst BSI with ostensibly communicable organisms (*S.aureus* and *S.pneumoniae*) remained stable. The observation of reduced Enterobacterales BSI immediately preceding and contemporaneous with public health measures, at a time when they would usually be increasing in incidence, requires urgent consideration [6, 7]. Given that Enterobacterales BSI could be considered non-communicable and likely endogenous in origin, incidence would be expected to remain similar allowing for seasonality. This observed reduction may reflect changes in presentation behaviour, with patients either self-isolating with ‘COVID-19 symptoms’ (e.g. fever), or avoiding healthcare due to fear of COVID-19 exposure. Certainly, emergency department attendances were lower than expected [9], in keeping with patients not presenting. Figures from England in 2018/19 show a 30-day mortality rate of 10.4 per 100,000 of the population and case fatality rate of 13.8% of cases [17] for Enterobacterales BSIs and there is concern that patients experiencing but not presenting with a BSI may have suffered fatalities in the community.

Widespread cancellation of elective surgical procedures offers an alternative explanation for the reduction in Enterobacterales BSIs. Gastrointestinal (GI) and urological procedures are a well-established source of Enterobacterales BSI [18, 19]. Furthermore, many patients presenting to hospital who fit the broad case definition for COVID-19 are rapidly started on broad-spectrum antimicrobials. As antimicrobials affect the detection rate of Enterobacterales from BCs [20], this may also be impacting our observed case rate.

The increased number of CoNS (non-CLABSI) BSIs seen during the pandemic may reflect increased rates of contamination. Contamination rates are reduced with increased phlebotomy expertise, but with staff performing procedures in unfamiliar PPE, the practicalities of venepuncture might prove more difficult. Additionally, more BCs are likely to be taken in patients with COVID-19 due to its febrile nature. This has been substantiated by Sepulveda et al. [21] who found a surge in BCs during March 2020, the majority of which were for SARS-CoV-2 positive patients, including repeated sampling. They found that CoNS accounted for 59.7% of positive BCs in these patients. Furthermore, data from Hughes et al. [22] has demonstrated infrequent confirmed secondary bacterial infection, approximately 3–6%. When planning for future COVID-19 peaks and/or other pandemics, these findings would support the need for dedicated teaching on aseptic non-touch techniques (ANTT) whilst in PPE, in an attempt to reduce contamination.

This could also potentially impact on antimicrobial stewardship and resistance [23]. Infection teams may recommend the addition of glycopeptides when Gram-positive cocci (GPC) are isolated from BCs, in the presence of a central line, whilst waiting full identification and sensitivities. To prevent unnecessary glycopeptide prescriptions, potential adverse drug events, antimicrobial resistance, and increased costs, it is important to have COVID-19 specific antimicrobial guidance reflecting the likelihood of increased BC contamination.

The absolute number of patients with CoNS CLABSI was also increased during April 2020 of the pandemic, which may reflect increased ITU capacity during this time, or alternatively, the challenges of managing patients within an ITU setting during the pandemic. For example, challenges of aseptic technique in full PPE as well as redeployment of staff from other areas to ITU and also, in patients with COVID-19, repositioning to try and improve oxygenation, increasing the risk of line displacement and/or contamination.

Our study has several limitations. We looked at total number of blood culture requests and so repeated samples from the same patients may have been included. Also, repeated positive BCs within a 14-day period were not de-duplicated for organisms other than CoNS CLABSI, which may have affected incidence. Also, the absolute number of patients with CoNS CLABSI was reported, but in order to determine if the rate of CoNS CLABSI was increased, we would have to look at ITU admission data over the same period. Furthermore, timing or setting of sampling (e.g. ED versus inpatient) was not assessed and thus whether Enterobacterales BSIs were community or hospital acquired. To gain finer resolution on the causes for the observed fall Enterobacterales BSI, it would be necessary to look at community and hospital acquired BSI related to elective GI/urological procedures over the same time frame. In addition, because this was an ecological analysis, we did not describe the epidemiology of patients including whether they had tested positive for COVID-19 or not, nor did we use a COVID-free hospital as a control.

## Conclusion

During the peak of the COVID-19 pandemic in London, significantly fewer Enterobacterales BSI occurred, alongside fewer documented emergency presentations. This potentially reflects reduced presentations of Enterobacterales BSI due to patients not presenting to secondary care. We suggest more nuanced public health messaging around self-isolation for febrile illnesses to ensure patients present to healthcare where necessary. High rates of CoNS BSI during the COVID-19 peak, likely due to contamination, might reflect an unintended consequence of PPE. We suggest increased training for ANTT procedures whilst using PPE to prepare for a second CoVID-19 wave or any future pandemics. We suggest specific antimicrobial guidelines for patients with COVID-19, not only accounting for fine resolution epidemiology on the frequency of bacterial co-infection, but also reflecting caution in reacting to GPCs in BC bottles.

## Supplementary Information

**Additional file 1.**

## Data Availability

The data analysed during the current study is available from the first author (SD; sarahdenny1@nhs.net) on reasonable request, as long as this meets local ethical and research governance criteria.

## Abbreviations

ANTT: Aseptic non-touch technique
ARIMA: Autoregressive integrated moving average
BC: Blood culture
BSI: Blood stream infection
CLABSI: Central line associated blood stream infection
CoNS: Coagulase negative Staphylococci
GI: Gastrointestinal
GNR-E: Gram negative rods – Enterobacterales
GNR-NE: Gram negative rods – Non Enterobacterales
GPC: Gram-positive cocci
MALDI-TOF: Matrix-assisted laser desorption/ionisation-time-of-flight
Non-CLABSI: Non-Central line associated blood stream infection
PPE: Personal protective equipment
SARS-CoV-2: Severe Acute Respiratory Syndrome coronavirus 2
S.pneumo: *Streptococcus pneumoniae*
UK: United Kingdom
