# Supplementary material for: Bacteraemia variation during the COVID-19 pandemic; a multi-centre UK secondary care ecological analysis
Source: BMC Infect Dis. 2021 Jun 11;21:556. doi: 10.1186/s12879-021-06159-8 (PMC8195453; doi:10.1186/s12879-021-06159-8)
Supplement: Supplementary file 1 — Additional file 1. [file 12879_2021_6159_MOESM1_ESM.pdf]

Date

Hospital	Group	2017	2018	2019	2020	Monthly Average (Based on 2018/19)	Standard dev	Hospital	Group	April	May	June
Hospital 1	CONS	99	113	102	59	8.958333333	3.495079357	Hospital 1	CONS	5	11	8
	CSS	54	94	72	17	6.916666667	4.009951389		CSS	5	7	6
	GNR-E	163	217	207	61	17.66666667	5.798550544		GNR-E	13	26	20
	GNR-NE	37	64	41	10	4.375	2.128296095		GNR-NE	5	2	5
	NSS	8	13	17	10	1.25	1.360147051		NSS		1	1
	Other	11	11	15	4	1.083333333	1.290994449		Other	1	2	
	Other (GPR)	17	19	18	6	1.541666667	1.286239389		Other (GPR)	2	2	3
	SA	33	63	57	11	5	2.827728316		SA	5	7	4
	Yeasts	9	16	10	4	1.083333333	0.864437822		Yeasts	1		1
Hospital 2	CONS	182	217	202	121	17.45833333	3.934011855	Hospital 2	CONS	17	16	22
	CSS	63	80	82	23	6.75	3.273742706		CSS	7	5	13
	GNR-E	192	238	259	70	20.70833333	5.287221401		GNR-E	25	19	16
	GNR-NE	52	57	47	23	4.333333333	2.31566812		GNR-NE	6	7	8
	NSS	38	18	34	10	2.166666667	1.788854382		NSS	2	10	4
	Other	13	17	19	4	1.5	1.151657844		Other			
	Other (GPR)	43	32	42	7	3.083333333	1.639635873		Other (GPR)	2	5	3
	SA	64	62	84	16	6.083333333	3.717370116		SA	8	1	3
	Yeasts	10	20	11	2	1.291666667	1.311403912		Yeasts	5		
Hospital 3	CONS	129	173	189	62	15.08333333	5.380453082	Hospital 3	CONS	18	13	25
	CSS	63	105	84	19	7.875	4.316928256		CSS	8	4	8
	GNR-E	211	203	190	45	16.375	3.6690539		GNR-E	24	21	27
	GNR-NE	51	89	120	24	8.708333333	5.120115205		GNR-NE	5	7	1
	NSS	27	34	28	13	2.583333333	2.500216441		NSS	2	2	3
	Other	7	15	6	3	0.875	0.960768923		Other		1	1
	Other (GPR)	14	43	19	5	2.583333333	2.322485607		Other (GPR)	1	2	
	SA	43	92	75	33	6.958333333	3.95678234		SA	8	7	6
	Yeasts	41	46	40	15	3.583333333	6.09785401		Yeasts	4	13	2
Hospital 4	CONS	142	163	170	122	13.875	4.875069676	Hospital 4	CONS	6	20	14
	CSS	57	96	64	24	6.666666667	3.509445189		CSS	6	7	6
	GNR-E	156	179	213	46	16.33333333	4.966554809		GNR-E	23	15	19
	GNR-NE	35	52	53	13	4.375	2.873485165		GNR-NE	2	5	4
	NSS	11	23	16	8	1.625	1.394538218		NSS	3	1	
	Other	9	13	11	5	1	0.730296743		Other	2	2	
	Other (GPR)	20	21	48	11	2.875	1.707127914		Other (GPR)	3	2	1
	SA	76	63	59	17	5.083333333	2.704117779		SA	9	9	7
	Yeasts	6	9	7		0.666666667	1.069044968		Yeasts	4		
Hospital 5	CONS	143	202	192	119	16.41666667	3.90001858	Hospital 5	CONS	16	18	11
	CSS	41	96	94	34	7.916666667	3.562078859		CSS	5	2	5
	GNR-E	236	353	320	89	28.04166667	6.265704906		GNR-E	21	22	18
	GNR-NE	51	48	39	11	3.625	2.081230823		GNR-NE	3	6	4
	NSS	21	27	35	12	2.583333333	1.465475707		NSS	1		1
	Other	15	24	24	10	2	1.124643114		Other	1	1	1
	Other (GPR)	28	46	34	6	3.333333333	1.830786349		Other (GPR)	1		3
	SA	38	47	66	21	4.708333333	2.136212957		SA		4	4
	Yeasts	3	7	13	6	0.833333333	1.250454463		Yeasts		1	1
**Across all sites**	CONS	695	868	855	483	71.79166667	11.09633377	All	CONS	62	78	80
	CSS	278	471	396	117	36.125	8.714368445		CSS	31	25	38
	GNR-E	958	1190	###	311	99.125	11.96484888		GNR-E	106	103	100
	GNR-NE	226	310	300	81	25.41666667	5.149222539		GNR-NE	21	27	22
	NSS	105	115	130	53	10.20833333	4.149166146		NSS	8	14	9
	Other	55	80	75	26	6.458333333	2.603996371		Other	4	6	2
	Other (GPR)	122	161	161	35	13.41666667	4.252024241		Other (GPR)	9	11	10
	SA	254	327	341	98	27.83333333	8.154308899		SA	30	28	24
	Yeasts	69	98	81	27	7.458333333	5.912250853		Yeasts	14	14	4

	Group	Number of blood cultures	% of total blood cultures
Blood cultures with no growth (N; % of all blood cultures)		109,144	91.27%
Blood cultures with isolate identified (N; % of all blood cultures)	CoNS all	2,855	2.39%
	- CLABSI	2466	2.06%
	- Non-CLABSI	341	0.29%
	- Unknown	48	0.04%
	S.pneumo	78	0.07%
	GNR-E	3,508	2.93%
	GNR-NE	790	0.66%
	Other	1975	1.65%
	S.aureus	988	0.83%
	Yeasts	246	0.21%

	2017										2018												2019		
July	August	September	October	November	December	January	February	March	April	May	June	July	August	September	October	November	December	January	February	March	April	May	June	July	August
11	8	11	16	13	16	7	9	8	9	13	12	6	7	9	5	8	20	11	6	4	8	8	9	12	14
6	4	8	2	8	8	11	5	3	9	4	4	4	7	9	17	10	11	7	10	3	6	3	3	1	2
24	19	13	21	17	10	15	10	15	13	12	19	26	22	15	36	15	19	26	15	13	18	14	18	21	14
6	2	4	6	7		6	9	3	9	3	5	5	5	7	6	4	2	4		6	4	7	4	2	4
1	1		1		3	1		1			3	2		1	2	3			1	1	2	6	1		
1	1	2	1	2	1						1		1	3		5	1		1	2		2	1	4	2
3	4		1		2		2	4	1			1	2	1	3	2	3	5				1	1		3
1	2	3	1	8	2	7	6	2	1	4	4	7	10	2	5	5	10	10	7	2	3	4	6	9	1
1	1		4		1		3	1		2	3	2	2	2	1				1		3		3		1
24	20	17	21	18	27	26	17	24	10	16	19	22	15	20	19	16	13	16	13	20	18	21	17	13	18
7	9	6	6	6	4	5	9	11	8	6	8	1	3	3	7	12	7	8	4	6	9	5	4	15	4
20	29	23	19	18	23	20	8	26	15	18	19	20	25	19	21	23	24	12	19	17	21	24	12	21	27
3	4	4	8	7	5	3	5	3	6	5	2	6	1	2	7	7	10	3	1	2	3	4	2	6	5
3	5	4		6	4	2	1			1	1	3	3	4	2		1	3	2	8	2	4	1	4	1
3	5	1	2	1	1		1	2	1	1	2	1			6	2	1	3	1	2		1	1	2	2
5	6	4	6	5	7	7	1	5	3	3	1	3	1	2	3	1	2	3	5	4	2	3	4	6	2
11	11	7	5	6	12	10	5	7	2	2	4	3	3	5	7	7	7	12	4	5	7	13	16	2	9
1	2	1		1		2	1	4					4	4	2	2	1	1		1	1	3	4		
6	25	13	9	12	8	9	26	25	7	9	7	11	14	21	20	14	10	19	19	18	12	11	12	15	16
2	7	6	6	5	17	12	15	6	11	10	8	1	10	10	5	9	8	6	1	4	13	10	7	4	13
12	20	28	28	29	22	14	13	18	14	25	13	18	12	19	20	21	16	16	11	12	13	15	17	21	19
7	1	3	5	10	12	9	3	9	2	18	4	3	14	5	12	7	3	7	18	5	17	7	7	10	5
3	4	6	1	1	5	4	2	1	3	2	9	1	6	2	3	1		3	10	1	2	3	1	1	3
		1	3	1		2	2			1		1	1	1	3	4			1				1		
4	1		1	1	4	4	4	1	1	2	4	1	10	6	7	1	2	2	3	3	1	2			3
1	2	9	2	4	4	16	6	4	3	13	10	6	5	3	1	14	11	3	4		6	10	11	6	7
5	3		11	3		2	23		1	4		11	1	1	1	1	1	1		2		2			12
18	14	18	18	15	19	20	14	24	10	11	7	4	13	13	19	16	12	12	17	10	14	9	7	14	20
4	5	4	12	6	7	16	9	14	7	12	2	9	6	4	8	9		7	4	3	7	9	6	3	6
17	17	19	19	19	8	12	24	10	14	17	13	14	12	22	10	19	12	13	16	15	15	20	17	19	19
3	3	2	5	6	5	9	3	4	3	3	3	3	4	13	3	1	3		1	3	5	3	4	4	9
	1	1	2	1	2		2	2	1	4	1		2	4	3	1	3		1	1		1	1	2	1
1	1	2	1			2		2		1	1	3	1	1		2			1		1	2		1	
2	1	2	3	1	5	2	3	3	1	2		2			6	1	1	3	5	2	3	7	4	5	5
7	11	3	17		13	5	4	5	5	8	12	4	2	2	5	8	3	3	6	8		4	6	2	6
	1		1			2			3	2			1			1			2		1				
10	14	16	14	22	22	17	16	24	13	14	15	17	17	20	14	15	20	12	23	15	18	11	17	11	9
3	7	4	4	6	5	8	8	3	5	7	6	14	9	8	5	11	12	13	6	3	5	7	5	7	4
25	38	26	25	31	30	20	28	33	34	36	21	26	27	37	29	39	23	24	25	28	24	27	20	31	24
7	4	8	2	9	8	2	6	5	3	1	3	10	1	6	3	5	3	3	3	4	1	4	3	1	2
10	1	2		2	4	2	1		1	2	2	5	4	2	3	2	3	1	3	5		2	3	2	4
1	1	1	6	1	2	5	2		1	2	1	2	3	2	2	1	3	1	2	2	2	1	3	2	2
1	6	3	6	5	3	4	4	4	2	3	3	2	6	5	3	3	7	5	5	1	1	1		7	3
8	4	7	3	3	5	3	4	2	3	2	8	1	6	4	8	4	2	5	7	7	6	6	4	3	6
1						2		1		1		1	1	1					2		3			1	
69	81	75	78	80	92	79	82	105	49	63	60	60	66	83	77	69	75	70	78	67	70	60	62	65	77
22	32	28	30	31	41	52	46	37	40	39	28	29	35	34	42	51	38	41	25	19	40	34	25	30	29
98	123	109	112	114	93	81	83	102	90	108	85	104	98	112	116	117	94	91	86	85	91	100	84	113	103
26	14	21	26	39	30	29	26	24	23	30	17	27	25	33	31	24	21	17	23	20	30	25	20	23	25
17	12	13	4	10	18	9	6	4	5	9	16	11	15	13	13	7	7	7	17	16	6	16	7	9	9
6	8	7	13	5	4	9	5	4	2	5	5	7	6	7	11	14	5	4	6	6	3	6	6	9	6
15	18	9	17	12	21	17	14	17	8	10	8	9	19	14	22	8	15	18	18	10	7	14	9	18	16
28	30	29	28	21	36	41	25	20	14	29	38	21	26	16	26	38	33	33	28	22	22	37	43	22	29
8	7	1	16	4	1	8	27	6	4	9	3	14	9	8	4	4	2	2	5	3	8	5	7	1	13

					2020		
September	October	November	December	January	February	March	April
6	6	7	11	8	14	24	13
8	6	14	9	9	3	1	4
22	16	19	11	31	10	8	12
2	3	4	1	4	4	1	1
1	3	1	1	5	1	2	2
2			1			2	2
1	2	4	1	2	3	1	
6		6	3	2	2	4	3
1		1			1	1	2
18	10	19	19	18	22	31	50
3	8	6	10	4	10	3	6
29	23	27	27	28	12	14	16
4	3	7	7	4	10	4	5
	1	3	5	2	3	4	1
2	2	2	1	2		1	1
4	1	4	4	4	1	1	1
3	2	5	6	4	6	3	3
1							2
18	21	11	17	12	19	15	16
6	2	2	16	8	3	6	2
16	22	15	13	20	7	9	9
7	6	15	16	3	8	5	8
1	1	2		8		4	1
	1	1	2	1			2
1	1	2	1		2	1	2
4	6	8	10	11	2	14	6
8		4	11	3		5	7
16	17	21	13	11	14	37	60
4	5	5	5	7	5	6	6
18	12	17	32	16	12	12	6
4	6	5	9	1	2	4	6
1	6	1	1	1	1	2	4
1	1	1	3	1	1	1	2
2	3	5	4	6	1	4	
2	6	11	5	2	1	9	5
4							
17	20	23	16	26	23	36	34
11	15	5	13	11	10	5	8
30	43	22	22	28	21	20	20
5	6	3	4	2	3	5	1
5	6	4		6	2	2	2
2	1	5	1	3	1	2	4
2	5	1	3		1	1	4
5	4	4	9	4	7	3	7
	2		5			1	5
75	74	81	76	75	92	143	173
32	36	32	53	39	31	21	26
115	116	100	105	123	62	63	63
22	24	34	37	14	27	19	21
8	17	11	7	22	7	14	10
7	5	9	8	7	2	6	11
10	12	16	13	12	8	8	7
20	18	34	33	23	18	33	24
14	2	5	16	3	1	7	16

Z. Scores

					2017										20
Hospital	Group	April	May	June	July	August	September	October	November	December	January	February	March	April	May	June
Hospital 1	CONS	-1.1325446	0.5841546	-0.274195	0.5841546	-0.274195	0.5841546	2.01473728	1.15638767	2.01473728	-0.5603116	0.01192152	-0.274195	0.01192152	1.15638767	0.87027113
	CSS	-0.4779775	0.02078163	-0.2285979	-0.2285979	-0.7273571	0.27016121	-1.2261163	0.27016121	0.27016121	1.01829996	-0.4779775	-0.9767367	0.5195408	-0.7273571	-0.7273571
	GNR-E	-0.8047988	1.43714076	0.40239941	1.09222698	0.22994252	-0.8047988	0.5748563	-0.1149713	-1.3221695	-0.459885	-1.3221695	-0.459885	-0.8047988	-0.9772557	0.22994252
	GNR-NE	0.29366215	-1.1159162	0.29366215	0.76352158	-1.1159162	-0.1761973	0.76352158	1.23338102	-2.055635	0.76352158	2.17309989	-0.6460567	2.17309989	-0.6460567	0.29366215
	NSS	-0.9190183	-0.1838037	-0.1838037	-0.1838037	-0.1838037	-0.9190183	-0.1838037	-0.9190183	1.28662559	-0.1838037	-0.9190183	-0.1838037	-0.9190183	-0.9190183	1.28662559
	Other	-0.0645497	0.71004695	-0.8391464	-0.0645497	-0.0645497	0.71004695	-0.0645497	0.71004695	-0.0645497	-0.8391464	-0.8391464	-0.8391464	-0.8391464	-0.8391464	-0.0645497
	Other (GPR)	0.35633595	0.35633595	1.1337962	1.1337962	1.91125645	-1.1985846	-0.4211243	-1.1985846	0.35633595	-1.1985846	0.35633595	1.91125645	-0.4211243	-1.1985846	-1.1985846
	SA	0	0.70728153	-0.3536408	-1.4145631	-1.0609223	-0.7072815	-1.4145631	1.06092229	-1.0609223	0.70728153	0.35364076	-1.0609223	-1.4145631	-0.3536408	-0.3536408
	Yeasts	-0.0964018	-1.253223	-0.0964018	-0.0964018	-0.0964018	-1.253223	3.37406184	-1.253223	-0.0964018	-1.253223	2.21724064	-0.0964018	-1.253223	1.06041944	2.21724064
Hospital 2	CONS	-0.1165053	-0.3706988	1.15446187	1.66284874	0.64607499	-0.1165053	0.90026843	0.13768811	2.42542906	2.17123562	-0.1165053	1.66284874	-1.8958594	-0.3706988	0.39188155
	CSS	0.07636519	-0.5345564	1.90912987	0.07636519	0.68728675	-0.2290956	-0.2290956	-0.2290956	-0.8400171	-0.5345564	0.68728675	1.29820831	0.38182597	-0.2290956	0.38182597
	GNR-E	0.8117055	-0.3231061	-0.8905119	-0.1339708	1.56824654	0.43343497	-0.3231061	-0.5122413	0.43343497	-0.1339708	-2.4035939	1.00084076	-1.0796471	-0.5122413	-0.3231061
	GNR-NE	0.71973469	1.1515755	1.58341631	-0.5757877	-0.1439469	-0.1439469	1.58341631	1.1515755	0.28789387	-0.5757877	0.28789387	-0.5757877	0.71973469	0.28789387	-1.0076286
	NSS	-0.0931695	4.37896646	1.02486449	0.4658475	1.58388148	1.02486449	-1.2112035	2.14289848	1.02486449	-0.0931695	-0.6521865	-1.2112035	-1.2112035	-0.6521865	-0.6521865
	Other	-1.3024702	-1.3024702	-1.3024702	1.30247018	3.03909709	-0.4341567	0.43415673	-0.4341567	-0.4341567	-1.3024702	-0.4341567	0.43415673	-0.4341567	-0.4341567	0.43415673
	Other (GPR)	-0.6607158	1.16895873	-0.0508243	1.16895873	1.77885024	0.55906722	1.77885024	1.16895873	2.38874175	2.38874175	-1.2706073	1.16895873	-0.0508243	-0.0508243	-1.2706073
	SA	0.51559748	-1.3674542	-0.8294394	1.32261962	1.32261962	0.2465901	-0.2914247	-0.0224173	1.59162701	1.05361224	-0.2914247	0.2465901	-1.0984468	-1.0984468	-0.560432
	Yeasts	2.82775833	-0.9849495	-0.9849495	-0.222408	0.54013361	-0.222408	-0.9849495	-0.222408	-0.9849495	0.54013361	-0.222408	2.06521676	-0.9849495	-0.9849495	-0.9849495
Hospital 3	CONS	0.5420857	-0.3872041	1.84309137	-1.6882097	1.84309137	-0.3872041	-1.1306359	-0.573062	-1.3164938	-1.1306359	2.02894933	1.84309137	-1.5023518	-1.1306359	-1.5023518
	CSS	0.02895577	-0.897629	0.02895577	-1.3609214	-0.2026904	-0.4343366	-0.4343366	-0.6659828	2.11377152	0.95554055	1.65047913	-0.4343366	0.72389436	0.49224816	0.02895577
	GNR-E	2.07819242	1.26054294	2.8958419	-1.1924055	0.98799312	3.16839172	3.16839172	3.44094155	1.53309277	-0.6473058	-0.9198557	0.44289347	-0.6473058	2.35074224	-0.9198557
	GNR-NE	-0.7242676	-0.3336513	-1.5055	-0.3336513	-1.5055	-1.1148838	-0.7242676	0.25227297	0.64288918	0.05696486	-1.1148838	0.05696486	-1.3101919	1.81473781	-0.9195757
	NSS	-0.2333131	-0.2333131	0.16665224	0.16665224	0.56661761	1.36654836	-0.6332785	-0.6332785	0.96658298	0.56661761	-0.2333131	-0.6332785	0.16665224	-0.2333131	2.56644447
	Other	-0.9107289	0.13010412	0.13010412	-0.9107289	-0.9107289	0.13010412	2.21177012	0.13010412	-0.9107289	1.17093712	1.17093712	-0.9107289	-0.9107289	0.13010412	-0.9107289
	Other (GPR)	-0.6817409	-0.2511677	-1.112314	0.60997866	-0.6817409	-1.112314	-0.6817409	-0.6817409	0.60997866	0.60997866	0.60997866	-0.6817409	-0.6817409	-0.2511677	0.60997866
	SA	0.26326105	0.01053044	-0.2422002	-1.5058532	-1.2531226	0.51599165	-1.2531226	-0.7476614	-0.7476614	2.2851059	-0.2422002	-0.7476614	-1.000392	1.52691408	0.76872226
	Yeasts	0.06833005	1.54425912	-0.2596542	0.23232217	-0.0956621	-0.5876384	1.21627488	-0.0956621	-0.5876384	-0.2596542	3.18418031	-0.5876384	-0.4236463	0.06833005	-0.5876384
Hospital 4	CONS	-1.6153615	1.2563923	0.02564066	0.84614175	0.02564066	0.84614175	0.84614175	0.23076593	1.05126703	1.2563923	0.02564066	2.07689339	-0.7948604	-0.5897352	-1.4102363
	CSS	-0.1899636	0.09498178	-0.1899636	-0.7598542	-0.4749089	-0.7598542	1.5197084	-0.1899636	0.09498178	2.6594897	0.66487243	2.08959905	0.09498178	1.5197084	-1.3297449
	GNR-E	1.34231211	-0.2684624	0.53692484	0.13423121	0.13423121	0.53692484	0.53692484	0.53692484	-1.6778901	-0.8725029	1.54365893	-1.2751965	-0.4698092	0.13423121	-0.6711561
	GNR-NE	-0.8265225	0.21750591	-0.1305035	-0.478513	-0.478513	-0.8265225	0.21750591	0.56551536	0.21750591	1.60954372	-0.478513	-0.1305035	-0.478513	-0.478513	-0.478513
	NSS	0.98598947	-0.448177	-1.1652603	-1.1652603	-0.448177	-0.448177	0.26890622	-0.448177	0.26890622	-1.1652603	0.26890622	0.26890622	-0.448177	1.70307272	-0.448177
	Other	1.36930639	1.36930639	-1.3693064	0	0	1.36930639	0	-1.3693064	-1.3693064	1.36930639	-1.3693064	1.36930639	-1.3693064	0	0
	Other (GPR)	0.0732224	-0.5125568	-1.098336	-0.5125568	-1.098336	-0.5125568	0.0732224	-1.098336	1.24478077	-0.5125568	0.0732224	0.0732224	-1.098336	-0.5125568	-1.6841152
	SA	1.44840831	1.44840831	0.70879556	0.70879556	2.18802107	-0.77043	4.40685933	-1.8798491	2.92763382	-0.0308172	-0.4006236	-0.0308172	-0.0308172	1.07860193	2.55782744
	Yeasts	3.11804782	-0.6236096	-0.6236096	-0.6236096	0.31180478	-0.6236096	0.31180478	-0.6236096	-0.6236096	1.24721913	-0.6236096	-0.6236096	2.18263348	1.24721913	-0.6236096
Hospital 5	CONS	-0.1068371	0.40598097	-1.3888823	-1.6452913	-0.6196552	-0.1068371	-0.6196552	1.43161711	1.43161711	0.14957194	-0.1068371	1.94443518	-0.8760642	-0.6196552	-0.3632461
	CSS	-0.8188102	-1.6610151	-0.8188102	-1.3802801	-0.2573404	-1.0995452	-1.0995452	-0.5380753	-0.8188102	0.02339458	0.02339458	-1.3802801	-0.8188102	-0.2573404	-0.5380753
	GNR-E	-1.1238427	-0.9642437	-1.6026396	-0.4854468	1.58933966	-0.3258479	-0.4854468	0.47214693	0.31254797	-1.2834417	-0.00665	0.79134485	0.95094382	1.27014174	-1.1238427
	GNR-NE	-0.3003031	1.14115166	0.18018184	1.62163656	0.18018184	2.10212147	-0.780788	2.58260638	2.10212147	-0.780788	1.14115166	0.66066675	-0.3003031	-1.2612729	-0.3003031
	NSS	-1.0804228	-1.7627951	-1.0804228	5.06092775	-1.0804228	-0.3980505	-1.7627951	-0.3980505	0.96669406	-0.3980505	-1.0804228	-1.7627951	-1.0804228	-0.3980505	-0.3980505
	Other	-0.889171	-0.889171	-0.889171	-0.889171	-0.889171	-0.889171	3.55668385	-0.889171	0	2.66751289	0	-1.7783419	-0.889171	0	-0.889171
	Other (GPR)	-1.2744979	-1.8207113	-0.1820711	-1.2744979	1.45656901	-0.1820711	1.45656901	0.91035563	-0.1820711	0.36414225	0.36414225	0.36414225	-0.7282845	-0.1820711	-0.1820711
	SA	-2.2040562	-0.3315837	-0.3315837	1.54088882	-0.3315837	1.0727707	-0.7997018	-0.7997018	0.13653445	-0.7997018	-0.3315837	-1.2678199	-0.7997018	-1.2678199	1.54088882
	Yeasts	-0.6664244	0.13328487	0.13328487	0.13328487	-0.6664244	-0.6664244	-0.6664244	-0.6664244	-0.6664244	0.93299412	-0.6664244	0.13328487	-0.6664244	0.13328487	-0.6664244
All	CONS	-0.8824236	0.55949411	0.73973382	-0.2515846	0.82985367	0.28913454	0.55949411	0.73973382	1.82117209	0.64961396	0.91997353	2.99273021	-2.0539817	-0.7923037	-1.0626633
	CSS	-0.5881092	-1.2766272	0.21516189	-1.6208863	-0.4733562	-0.9323682	-0.7028622	-0.5881092	0.55942092	1.82170402	1.13318596	0.10040888	0.44466791	0.3299149	-0.9323682
	GNR-E	0.57459982	0.32386535	0.07313089	-0.0940254	1.99542846	0.82533429	1.07606875	1.24322506	-0.5119162	-1.5148541	-1.3476978	0.2402872	-0.7626507	0.74175613	-1.1805414
	GNR-NE	-0.8577347	0.30748979	-0.6635306	0.11328571	-2.2171632	-0.8577347	0.11328571	2.63793869	0.89010201	0.69589794	0.11328571	-0.2751224	-0.4693265	0.89010201	-1.634551
	NSS	-0.5322355	0.91383823	-0.2912232	1.63687508	0.43181367	0.67282595	-1.4962846	-0.0502109	1.87788736	-0.2912232	-1.01426	-1.4962846	-1.2552723	-0.2912232	1.3958628
	Other	-0.9440617	-0.1760115	-1.712112	-0.1760115	0.59203872	0.2080136	2.51216428	-0.5600366	-0.9440617	0.97606383	-0.5600366	-0.9440617	-1.712112	-0.5600366	-0.5600366
	Other (GPR)	-1.038721	-0.5683567	-0.8035388	0.37237166	1.07791797	-1.038721	0.84273587	-0.3331746	1.78346428	0.84273587	0.13718956	0.84273587	-1.2739031	-0.8035388	-1.2739031
	SA	0.26570819	0.02043909	-0.4700991	0.02043909	0.26570819	0.14307364	0.02043909	-0.8380028	1.00151549	1.61468824	-0.3474646	-0.9606373	-1.6964446	0.14307364	1.24678459
	Yeasts	1.10645959	1.10645959	-0.5849436	0.09161767	-0.0775226	-1.0923646	1.44474023	-0.5849436	-1.0923646	0.09161767	3.30528375	-0.246663	-0.5849436	0.26075799	-0.7540839

18											2019								2020		
July	August	September	October	November	December	January	February	March	April	May	June	July	August	September	October	November	December	January	February	March	April
-0.8464281	-0.5603116	0.01192152	-1.1325446	-0.274195	3.15920342	0.5841546	-0.8464281	-1.4186612	-0.274195	-0.274195	0.01192152	0.87027113	1.4425042	-0.8464281	-0.8464281	-0.5603116	0.5841546	-0.274195	1.4425042	4.30366957	1.15638767
-0.7273571	0.02078163	0.5195408	2.51457745	0.76892038	1.01829996	0.02078163	0.76892038	-0.9767367	-0.2285979	-0.9767367	-0.9767367	-1.4754959	-1.2261163	0.27016121	-0.2285979	1.7664387	0.5195408	0.5195408	-0.9767367	-1.4754959	-0.7273571
1.43714076	0.7473132	-0.459885	3.16170967	-0.459885	0.22994252	1.43714076	-0.459885	-0.8047988	0.05748563	-0.6323419	0.05748563	0.5748563	-0.6323419	0.7473132	-0.2874282	0.22994252	-1.1497126	2.29942522	-1.3221695	-1.6670833	-0.9772557
0.29366215	0.29366215	1.23338102	0.76352158	-0.1761973	-1.1159162	-0.1761973	-2.055635	0.76352158	-0.1761973	1.23338102	-0.1761973	-1.1159162	-0.1761973	-1.1159162	-0.6460567	-0.1761973	-1.5857756	-0.1761973	-0.1761973	-1.5857756	-1.5857756
0.55141097	-0.9190183	-0.1838037	0.55141097	1.28662559	-0.9190183	-0.9190183	-0.1838037	-0.1838037	0.55141097	3.49226945	-0.1838037	-0.9190183	-0.9190183	-0.1838037	1.28662559	-0.1838037	-0.1838037	2.75705483	-0.1838037	0.55141097	0.55141097
-0.8391464	-0.0645497	1.48464362	-0.8391464	3.03383695	-0.0645497	-0.8391464	-0.0645497	0.71004695	-0.8391464	0.71004695	-0.0645497	2.25924029	0.71004695	0.71004695	-0.8391464	-0.8391464	-0.0645497	-0.8391464	-0.8391464	0.71004695	0.71004695
-0.4211243	0.35633595	-0.4211243	1.1337962	0.35633595	1.1337962	2.68871671	-1.1985846	-1.1985846	-1.1985846	-0.4211243	-0.4211243	-1.1985846	1.1337962	-0.4211243	0.35633595	1.91125645	-0.4211243	0.35633595	1.1337962	-0.4211243	-1.1985846
0.70728153	1.76820382	-1.0609223	0	0	1.76820382	1.76820382	0.70728153	-1.0609223	-0.7072815	-0.3536408	0.35364076	1.41456305	-1.4145631	0.35364076	-1.7682038	0.35364076	-0.7072815	-1.0609223	-1.0609223	-0.3536408	-0.7072815
1.06041944	1.06041944	1.06041944	-0.0964018	-1.253223	-1.253223	-1.253223	-0.0964018	-1.253223	2.21724064	-1.253223	2.21724064	-1.253223	-0.0964018	-0.0964018	-1.253223	-0.0964018	-1.253223	-1.253223	-0.0964018	-0.0964018	1.06041944
1.15446187	-0.6248922	0.64607499	0.39188155	-0.3706988	-1.1332791	-0.3706988	-1.1332791	0.64607499	0.13768811	0.90026843	-0.1165053	-1.1332791	0.13768811	0.13768811	-1.8958594	0.39188155	0.39188155	0.13768811	1.15446187	3.44220281	8.27187814
-1.7563995	-1.1454779	-1.1454779	0.07636519	1.60366909	0.07636519	0.38182597	-0.8400171	-0.2290956	0.68728675	-0.5345564	-0.8400171	2.52005143	-0.8400171	-1.1454779	0.38182597	-0.2290956	0.99274753	-0.8400171	0.99274753	-1.1454779	-0.2290956
-0.1339708	0.8117055	-0.3231061	0.05516445	0.43343497	0.62257023	-1.6470529	-0.3231061	-0.7013766	0.05516445	0.62257023	-1.6470529	0.05516445	1.18997602	1.56824654	0.43343497	1.18997602	1.18997602	1.37911128	-1.6470529	-1.2687824	-0.8905119
0.71973469	-1.4394694	-1.0076286	1.1515755	1.1515755	2.44709793	-0.5757877	-1.4394694	-1.0076286	-0.5757877	-0.1439469	-1.0076286	0.71973469	0.28789387	-0.1439469	-0.5757877	1.1515755	1.1515755	-0.1439469	2.44709793	-0.1439469	0.28789387
0.4658475	0.4658475	1.02486449	-0.0931695	-1.2112035	-0.6521865	0.4658475	-0.0931695	3.26093247	-0.0931695	1.02486449	-0.6521865	1.02486449	-0.6521865	-1.2112035	-0.6521865	0.4658475	1.58388148	-0.0931695	0.4658475	1.02486449	-0.6521865
-0.4341567	-1.3024702	-1.3024702	3.90741054	0.43415673	-0.4341567	1.30247018	-0.4341567	0.43415673	-1.3024702	-0.4341567	-0.4341567	0.43415673	0.43415673	0.43415673	0.43415673	0.43415673	-0.4341567	0.43415673	-1.3024702	-0.4341567	-0.4341567
-0.0508243	-1.2706073	-0.6607158	-0.0508243	-1.2706073	-0.6607158	-0.0508243	1.16895873	0.55906722	-0.6607158	-0.0508243	0.55906722	1.77885024	-0.6607158	0.55906722	-1.2706073	0.55906722	0.55906722	0.55906722	-1.2706073	-1.2706073	-1.2706073
-0.8294394	-0.8294394	-0.2914247	0.2465901	0.2465901	0.2465901	1.59162701	-0.560432	-0.2914247	0.2465901	1.86063439	2.66765653	-1.0984468	0.78460486	-0.8294394	-1.0984468	-0.2914247	-0.0224173	-0.560432	-0.0224173	-0.8294394	-0.8294394
-0.9849495	2.06521676	2.06521676	0.54013361	0.54013361	-0.222408	-0.222408	-0.9849495	-0.222408	-0.222408	1.30267518	2.06521676	-0.9849495	-0.9849495	-0.222408	-0.9849495	-0.9849495	-0.9849495	-0.9849495	-0.9849495	-0.9849495	0.54013361
-0.75892	-0.2013461	1.09965956	0.91380161	-0.2013461	-0.9447779	0.72794365	0.72794365	0.5420857	-0.573062	-0.75892	-0.573062	-0.0154882	0.17036979	0.5420857	1.09965956	-0.75892	0.35622774	-0.573062	0.72794365	-0.0154882	0.17036979
-1.5925676	0.49224816	0.49224816	-0.6659828	0.26060197	0.02895577	-0.4343366	-1.5925676	-0.897629	1.18718674	0.49224816	-0.2026904	-0.897629	1.18718674	-0.4343366	-1.3609214	-1.3609214	1.88212533	0.02895577	-1.1292752	-0.4343366	-1.3609214
0.44289347	-1.1924055	0.71544329	0.98799312	1.26054294	-0.1022062	-0.1022062	-1.4649553	-1.1924055	-0.9198557	-0.374756	0.17034364	1.26054294	0.71544329	-0.1022062	1.53309277	-0.374756	-0.9198557	0.98799312	-2.5551546	-2.010055	-2.010055
-1.1148838	1.03350539	-0.7242676	0.64288918	-0.3336513	-1.1148838	-0.3336513	1.81473781	-0.7242676	1.61942971	-0.3336513	-0.3336513	0.25227297	-0.7242676	-0.3336513	-0.5289595	1.2288135	1.4241216	-1.1148838	-0.1383432	-0.7242676	-0.1383432
-0.6332785	1.36654836	-0.2333131	0.16665224	-0.6332785	-1.0332439	0.16665224	2.96640985	-0.6332785	-0.2333131	0.16665224	-0.6332785	-0.6332785	0.16665224	-0.6332785	-0.6332785	-0.2333131	-1.0332439	2.1664791	-1.0332439	0.56661761	-0.6332785
0.13010412	0.13010412	0.13010412	2.21177012	3.25260312	-0.9107289	-0.9107289	0.13010412	-0.9107289	-0.9107289	-0.9107289	0.13010412	-0.9107289	-0.9107289	-0.9107289	0.13010412	0.13010412	1.17093712	0.13010412	-0.9107289	-0.9107289	1.17093712
-0.6817409	3.19341771	1.47112501	1.90169819	-0.6817409	-0.2511677	-0.2511677	0.17940549	0.17940549	-0.6817409	-0.2511677	-1.112314	-1.112314	0.17940549	-0.6817409	-0.6817409	-0.2511677	-0.6817409	-1.112314	-0.2511677	-0.6817409	-0.2511677
-0.2422002	-0.4949308	-1.000392	-1.5058532	1.77964469	1.02145287	-1.000392	-0.7476614	-1.7585838	-0.2422002	0.76872226	1.02145287	-0.2422002	0.01053044	-0.7476614	-0.2422002	0.26326105	0.76872226	1.02145287	-1.2531226	1.77964469	-0.2422002
1.21627488	-0.4236463	-0.4236463	-0.4236463	-0.4236463	-0.4236463	-0.4236463	-0.5876384	-0.2596542	-0.5876384	-0.2596542	-0.5876384	-0.5876384	1.380267	0.72429853	-0.5876384	0.06833005	1.21627488	-0.0956621	-0.5876384	0.23232217	0.56030641
-2.0256121	-0.1794846	-0.1794846	1.05126703	0.43589121	-0.3846099	-0.3846099	0.64101648	-0.7948604	0.02564066	-0.9999857	-1.4102363	0.02564066	1.2563923	0.43589121	0.64101648	1.46151757	-0.1794846	-0.5897352	0.02564066	4.74352195	9.46140323
0.66487243	-0.1899636	-0.7598542	0.3799271	0.66487243	-1.8996355	0.09498178	-0.7598542	-1.0447995	0.09498178	0.66487243	-0.1899636	-1.0447995	-0.1899636	-0.7598542	-0.4749089	-0.4749089	-0.4749089	0.09498178	-0.4749089	-0.1899636	-0.1899636
-0.4698092	-0.8725029	1.14096529	-1.2751965	0.53692484	-0.8725029	-0.6711561	-0.0671156	-0.2684624	-0.2684624	0.73827166	0.13423121	0.53692484	0.53692484	0.33557803	-0.8725029	0.13423121	3.15443346	-0.0671156	-0.8725029	-0.8725029	-2.0805838
-0.478513	-0.1305035	3.00158153	-0.478513	-1.1745319	-0.478513	-1.5225414	-1.1745319	-0.478513	0.21750591	-0.478513	-0.1305035	-0.1305035	1.60954372	-0.1305035	0.56551536	0.21750591	1.60954372	-1.1745319	-0.8265225	-0.1305035	0.56551536
-1.1652603	0.26890622	1.70307272	0.98598947	-0.448177	0.98598947	-1.1652603	-0.448177	-0.448177	-1.1652603	-0.448177	-0.448177	0.26890622	-0.448177	-0.448177	3.13723923	-0.448177	-0.448177	-0.448177	-0.448177	0.26890622	1.70307272
2.73861279	0	0	-1.3693064	1.36930639	-1.3693064	-1.3693064	0	-1.3693064	0	1.36930639	-1.3693064	0	-1.3693064	0	0	0	2.73861279	0	0	0	1.36930639
-0.5125568	-1.6841152	-1.6841152	1.83055996	-1.098336	-1.098336	0.0732224	1.24478077	-0.5125568	0.0732224	2.41633914	0.65900158	1.24478077	1.24478077	-0.5125568	0.0732224	1.24478077	0.65900158	1.83055996	-1.098336	0.65900158	-1.6841152
-0.4006236	-1.1402363	-1.1402363	-0.0308172	1.07860193	-0.77043	-0.77043	0.33898918	1.07860193	-1.8798491	-0.4006236	0.33898918	-1.1402363	0.33898918	-1.1402363	0.33898918	2.18802107	-0.0308172	-1.1402363	-1.5100427	1.44840831	-0.0308172
-0.6236096	0.31180478	-0.6236096	-0.6236096	0.31180478	-0.6236096	-0.6236096	1.24721913	-0.6236096	0.31180478	-0.6236096	-0.6236096	-0.6236096	-0.6236096	3.11804782	-0.6236096	-0.6236096	-0.6236096	-0.6236096	-0.6236096	-0.6236096	-0.6236096
0.14957194	0.14957194	0.91879904	-0.6196552	-0.3632461	0.91879904	-1.1324732	1.68802615	-0.3632461	0.40598097	-1.3888823	0.14957194	-1.3888823	-1.9017003	0.14957194	0.91879904	1.68802615	-0.1068371	2.45725325	1.68802615	5.0213436	4.50852553
1.70780423	0.30412952	0.02339458	-0.8188102	0.8655994	1.14633434	1.42706929	-0.5380753	-1.3802801	-0.8188102	-0.2573404	-0.8188102	-0.2573404	-1.0995452	0.8655994	1.98853917	-0.8188102	1.42706929	0.8655994	0.58486446	-0.8188102	0.02339458
-0.3258479	-0.1662489	1.4297407	0.15294901	1.74893863	-0.8046448	-0.6450458	-0.4854468	-0.00665	-0.6450458	-0.1662489	-1.2834417	0.47214693	-0.6450458	0.31254797	2.38733448	-0.9642437	-0.9642437	-0.00665	-1.1238427	-1.2834417	-1.2834417
3.06309129	-1.2612729	1.14115166	-0.3003031	0.66066675	-0.3003031	-0.3003031	-0.3003031	0.18018184	-1.2612729	0.18018184	-0.3003031	-1.2612729	-0.780788	0.66066675	1.14115166	-0.3003031	0.18018184	-0.780788	-0.3003031	0.66066675	-1.2612729
1.64906634	0.96669406	-0.3980505	0.28432178	-0.3980505	0.28432178	-1.0804228	0.28432178	1.64906634	-1.7627951	-0.3980505	0.28432178	-0.3980505	0.96669406	1.64906634	2.33143862	0.96669406	-1.7627951	2.33143862	-0.3980505	-0.3980505	-0.3980505
0	0.88917096	0	0	-0.889171	0.88917096	-0.889171	0	0	0	-0.889171	0.88917096	0	0	0	-0.889171	2.66751289	-0.889171	0.88917096	-0.889171	0	1.77834192
-0.7282845	1.45656901	0.91035563	-0.1820711	-0.1820711	2.00278239	0.91035563	0.91035563	-1.2744979	-1.2744979	-1.2744979	-1.8207113	2.00278239	-0.1820711	-0.7282845	0.91035563	-1.2744979	-0.1820711	-1.8207113	-1.2744979	-1.2744979	0.36414225
-1.735938	0.60465258	-0.3315837	1.54088882	-0.3315837	-1.2678199	0.13653445	1.0727707	1.0727707	0.60465258	0.60465258	-0.3315837	-0.7997018	0.60465258	0.13653445	-0.3315837	-0.3315837	2.00900695	-0.3315837	1.0727707	-0.7997018	1.0727707
0.13328487	0.13328487	0.13328487	-0.6664244	-0.6664244	-0.6664244	-0.6664244	0.93299412	-0.6664244	1.73270337	-0.6664244	-0.6664244	0.13328487	-0.6664244	-0.6664244	0.93299412	-0.6664244	3.33212187	-0.6664244	-0.6664244	0.13328487	3.33212187
-1.0626633	-0.5219442	1.01009338	0.46937425	-0.2515846	0.28913454	-0.1614647	0.55949411	-0.4318243	-0.1614647	-1.0626633	-0.8824236	-0.612064	0.46937425	0.28913454	0.19901468	0.82985367	0.37925439	0.28913454	1.82117209	6.41728474	9.12088041
-0.8176152	-0.1290971	-0.2438501	0.67417393	1.70695101	0.21516189	0.55942092	-1.2766272	-1.9651453	0.44466791	-0.2438501	-1.2766272	-0.7028622	-0.8176152	-0.4733562	-0.0143441	-0.4733562	1.93645703	0.3299149	-0.5881092	-1.7356393	-1.1618742
0.40744351	-0.0940254	1.07606875	1.41038137	1.49395953	-0.428338	-0.6790725	-1.0969633	-1.1805414	-0.6790725	0.07313089	-1.2641196	1.15964691	0.32386535	1.32680322	1.41038137	0.07313089	0.49102166	1.99542846	-3.102839	-3.0192609	-3.0192609
0.30748979	-0.0809184	1.47271424	1.08430609	-0.2751224	-0.8577347	-1.634551	-0.4693265	-1.0519387	0.89010201	-0.0809184	-1.0519387	-0.4693265	-0.0809184	-0.6635306	-0.2751224	1.66691831	2.24953054	-2.2171632	0.30748979	-1.2461428	-0.8577347
0.19080139	1.15485052	0.67282595	0.67282595	-0.7732477	-0.7732477	-0.7732477	1.63687508	1.3958628	-1.01426	1.3958628	-0.7732477	-0.2912232	-0.2912232	-0.5322355	1.63687508	0.19080139	-0.7732477	2.84193649	-0.7732477	0.91383823	-0.0502109
0.2080136	-0.1760115	0.2080136	1.74411405	2.89618939	-0.5600366	-0.9440617	-0.1760115	-0.1760115	-1.3280868	-0.1760115	-0.1760115	0.97606383	-0.1760115	0.2080136	-0.5600366	0.97606383	0.59203872	0.2080136	-1.712112	-0.1760115	1.74411405
-1.038721	1.31310007	0.13718956	2.01864638	-1.2739031	0.37237166	1.07791797	1.07791797	-0.8035388	-1.5090852	0.13718956	-1.038721	1.07791797	0.60755376	-0.8035388	-0.3331746	0.60755376	-0.0979925	-0.3331746	-1.2739031	-1.2739031	-1.5090852
-0.8380028	-0.22483	-1.4511755	-0.22483	1.24678459	0.63361184	0.63361184	0.02043909	-0.7153682	-0.7153682	1.12415004	1.85995734	-0.7153682	0.14307364	-0.9606373	-1.2059064	0.75624639	0.63361184	-0.5927337	-1.2059064	0.63361184	-0.4700991
1.10645959	0.26075799	0.09161767	-0.5849436	-0.5849436	-0.9232242	-0.9232242	-0.4158033	-0.7540839	0.09161767	-0.4158033	-0.0775226	-1.0923646	0.93731927	1.10645959	-0.9232242	-0.4158033	1.44474023	-0.7540839	-1.0923646	-0.0775226	1.44474023
